# Mitochondrial Fragmentation Induced by the CFTR Modulators Lumacaftor and Ivacaftor in Immortalized Cystic Fibrosis Cell Lines

**DOI:** 10.3390/cells14201601

**Published:** 2025-10-15

**Authors:** Camila Dib, Pablo A. Iglesias González, María de los Ángeles Aguilar, Guillermo L. Taminelli, Tatiana Limpias del Valle, Nadia E. Nuñez, Analía G. Karadayian, Tomás A. Santa-Coloma, Ángel G. Valdivieso

**Affiliations:** 1 Laboratory of Cellular and Molecular Biology, Institute for Biomedical Research (BIOMED), School of Medical Sciences, Pontifical Catholic University of Argentina (UCA), National Scientific and Technical Research Council of Argentina (CONICET), Alicia Moreau de Justo 1600, Buenos Aires 1107, Argentina; cdib@uca.edu.ar (C.D.); pabloiglesias@uca.edu.ar (P.A.I.G.); mdelosangelesaguilar@uca.edu.ar (M.d.l.Á.A.); tlimpiasdelvalle@uade.edu.ar (T.L.d.V.); nadinunez@uade.edu.ar (N.E.N.); analiakaradayian@uca.edu.ar (A.G.K.); tomas_santacoloma@uca.edu.ar (T.A.S.-C.); 2 School of Engineering and Agrarian Sciences (FICA), Pontifical Catholic University of Argentina (UCA), Buenos Aires 1107, Argentina; guillermo_taminelli@uca.edu.ar

**Keywords:** CFTR, lumacaftor, ivacaftor, mitochondrial morphology

## Abstract

Cystic fibrosis (CF) is an autosomal recessive disease caused by mutations in the CFTR gene, which encodes a cAMP-activated chloride channel essential for epithelial function. Beyond its canonical role, evidence suggests CFTR also influences mitochondrial function. Previous studies have identified CFTR- and Cl-dependent genes, including *MTND4* and *CISD1*, which are downregulated in CF cells and play a critical role in mitochondrial function. CF cells exhibit altered mitochondrial complex I (mCx-I) activity and impaired electron transport chain function, although the underlying mechanisms remain unclear. In this study, the impact of the CFTR modulators lumacaftor (VX-809) and ivacaftor (VX-770) on mitochondrial morphology and function was investigated in heterozygous ΔF508/W1282X CF IB3-1 cells. Combined treatment with VX-809 (10 μM, CFTR corrector) and VX-770 (0.1 μM, CFTR potentiator) induced a fragmented mitochondrial morphology in both CF and CF expressing wt-CFTR cells, without affecting cell viability or mitochondrial membrane potential (ΔΨm). While individual treatments differentially modulated ROS production and ΔΨm, these effects were not statistically significant under combined treatment. These results highlight a previously unrecognized role for CFTR modulators in shaping mitochondrial morphology. A better understanding of these effects may reveal novel mechanisms underlying the regulation of mitochondrial structure and function.

## 1. Introduction

Cystic fibrosis (CF) is an autosomal recessive disorder caused by mutations in the CFTR gene (cystic fibrosis transmembrane conductance regulator) [1]. The encoded protein, CFTR, is an integral membrane glycoprotein located in the apical region of epithelial cells [2] and functions as a cAMP-activated chloride channel [3]. The high evolutionary conservation of the CFTR gene among species, along with the severe consequences of its mutations in CF, underscores the channel’s critical role in mammalian cells [4].

Before the discovery of CFTR mutation, mitochondrial abnormalities in CF had been reported [5,6,7,8,9,10]. However, after the identification of CFTR, research efforts were primarily focused on its structure and function. Recently, studies have revealed a link between CFTR dysfunction and mitochondrial impairment [11,12,13,14,15,16,17,18,19]. In previous work, we have identified CFTR-dependent genes, including MTND4 and CISD1 (mitoNEET), which are downregulated in CF cells and encode key mitochondrial proteins [20,21]. Notably, MTND4 [22] and CISD1 [21] play critical roles in mitochondrial function. MTND4 is crucial for mitochondrial complex I (mCx-I) assembly [23,24,25], and its deficiency correlates with reduced mCx-I activity in CF cells. This agrees with reports of altered mCx-I-III activity and electron transport chain (ETC) dysfunction in CF [12,15,18].

Recently, we reported that the pharmacological inhibition of CFTR induces an increase in the mitochondrial fission state, suggesting that CFTR activity may modulate mitochondrial dynamics [26]. It is well established that mitochondrial morphology and function depend on the balance between fission (mediated by DRP1, FIS1, and accessory proteins) [27] and fusion (controlled by MFN1/2 and OPA1) [28,29,30], along with mitophagy for quality control and turnover [31,32]. Disruption of the fission/fusion balance may increase susceptibility to bacterial and viral infections by impairing critical immune and metabolic responses [33,34,35]. To what extent mitochondrial dynamics may influence susceptibility to infections in CF remains an unexplored issue.

Mitochondrial dynamics regulate key antimicrobial defenses by modulating mitophagy, reactive oxygen species (ROS) production, and activation of the NLRP3 inflammasome [36]. During bacterial infections, pathogens have been reported to manipulate mitochondrial dynamics by inducing either fission or fusion [37,38]. Moreover, several studies suggest an important role of mitochondrial dynamics during viral infections [39]. The MAVS protein (localized to the mitochondrial outer membrane) and the STING protein (localized to the endoplasmic reticulum), both involved in the formation of an antiviral signaling complex, have been reported to be associated with the mitochondrial fusion proteins MFN1/2 to carry out their functions [35]. Thus, the role of CFTR in the regulation of mitochondrial morphology could be highly relevant for understanding defects in innate immunity associated with CF or infectious diseases.

The mechanisms linking CFTR dysfunction to mitochondrial impairments remain unclear. We hypothesized that CFTR activity (rather than its mere presence/absence at the plasma membrane) regulates mitochondrial fission/fusion dynamics, potentially driving CF-related mitochondrial defects [12,15,16]. Due to its structural and regulatory complexity, CFTR may act as a molecular signaling hub rather than merely as a conventional ion channel. Several studies suggest that CFTR interacts with various intracellular processes through associations with other proteins [40,41,42], facilitating the clustering of ion channels into microdomains at the cell surface and thereby promoting the assembly of signaling complexes at the plasma membrane [43]. Recently, it has been reported that CFTR can be activated by Ca^2+^ independently of PKA or PKC, through calmodulin [44,45,46]. These findings suggest a potential role for CFTR as a signaling center capable of regulating numerous cellular processes, including the expression of specific genes (CFTR-dependent genes) [21,22,47,48].

In recent years, CF treatment has focused on the use of correctors and potentiators, such as lumacaftor, ivacaftor, tezacaftor and elexacaftor (collectively known as modulators), to improve CFTR function [49,50,51,52,53]. While these compounds show promise in restoring chloride channel activity, their effects on mitochondrial metabolism remain poorly understood [54]. In this study, we investigated the impact of CFTR modulators on mitochondrial morphology and function using the first FDA-approved corrector, lumacaftor (VX-809), and the potentiator ivacaftor (VX-770) [55,56].

## 2. Materials and Methods

### 2.1. Reagents

Lumacaftor (VX-809, 3-(6-(1-(2,2-difluorobenzo[d][1,3]dioxol-5-yl)cyclopropanecarboxamido)-3-methylpyridin-2-yl)benzoic acid, Cat. N° HY-13262), ivacaftor (VX-770, N-(2,4-di-tert-butyl-5-hydroxyphenyl)-4-oxo-1,4-dihydroquinoline-3-carboxamide), Cat. N° HY-13017), protease inhibitor cocktail (Cat. N° HY-K0010), MQAE (N-(ethoxycarbonylmethyl)-6-methoxyquinolinium bromide, Cat. N° HY-D0090), Nigericin (Cat. N° HY-127019) and phosphatase inhibitors (Cat. N° HY-K0022), were purchased from MedChemExpress (Monmouth Junction, NJ, USA). The stock solutions of inhibitors were prepared at 1000X in culture-grade DMSO. DCFH-DA (2′,7′-Dichlorodihydrofluorescein Diacetate, Invitrogen, Cat. N° D-399), MitoTracker Orange CMTMRos ((9-(4-(chloromethyl)phenyl)-6-(dimethylamino)xanthen-3-ylidene)-dimethylazanium;chloride, Cat. N° M7510, Invitrogen), MitoSOX Red mitochondrial superoxide indicator (6-(3,8-diamino-6-phenyl-6H-phenanthridin-5-yl)hexyl-triphenylphosphanium iodide, Cat. N° M36008, Invitrogen), JC-1 (5,5′,6,6′-tetrachloro-1,1′,3,3′-tetraethylbenzimidazolylcarbocyanine iodide; Invitrogen, Cat. N° T3168) and TMRE (Tetramethylrhodamine, ethyl ester, perchlorate, Invitrogen, Cat. N° T669) were purchased from Thermo Fisher Scientific Inc. (Waltham, MA, USA). ATP (Cat. N° FLAAS), FCCP (Cat. N° C2920), luciferase (Cat. N° L8507), D-luciferine (Cat. N° L9504), Mito-TEMPO (Cat. N° SML0737, 2-(2,2,6,6-Tetramethylpiperidin-1-oxyl-4-ylamino)-2-oxoethyl)triphenylphosphonium chloride), NADH (Cat. N°, N8129), N-acetyl-L-cysteine (NAC) (Cat. N° A7250), tributyltin chloride (Cat. N° T50202), Pepstatin A (Cat. N° P5318), Leupeptin (Cat. N° L5793), PMSF (Cat. N° P7626), dibutyryl cAMP sodium salt (Cat. N° 28745-M), 3-isobutyl-1-methylxanthine (IBMX) (Cat. N° I7018), (-)-isoproterenol hydrochloride (Cat. N° I6504), and valinomycin (Cat. N° V0627) were purchased from Sigma-Aldrich (St. Louis, MO, USA).

### 2.2. Cultured Cells

IB3-1 (RRID: CVCL_0338), IB3/S9 (RRID: CVCL_4461) and IB3/C38 (RRID: CVCL_4462) cell lines were purchased from ATCC (now available at John Hopkins University). IB3-1 cells are bronchial epithelial cells derived from a CF patient that exhibited the most frequent mutation ΔF508 in one allele [57,58]. These cells also have the nonsense mutation W1282X in the second allele, which induces a severe disease by itself [59]. The IB3-1 cells have been immortalized using the hybrid adenovirus adeno-12-SV40 (ΔF508/W1282X [57]). S9 and C38 are IB3-1 cells, both transduced with adeno-associated viral vectors to stably expresses CFTR. S9 expresses wt-CFTR [60], while C38 express a functional truncated version (del-1-119-CFTR) [61]. All cell lines were cultured and maintained in DMEM/F12 with 15 mM HCO_3_^−^ and 15 mM HEPES with 5% FBS, 100 U/mL penicillin and 100 μg/mL streptomycin (Life Technologies, GIBCO BRL, Rockville, MD, USA). Cultures were grown in a humidified atmosphere at 37 °C with 5% CO_2_. Cells were incubated with serum-free DMEM/F12 for 24 h before the experiments, as previously described [26].

### 2.3. Treatments

Cells were treated with different combinations of CF modulators. Upon reaching 70% confluence, cells were incubated in serum-free DMEM/F12 24 h before experiments. Subsequently, cells were treated for 48 h with the respective treatments. Five different treatment combinations were used, which included: DMSO (vehicle) for 48 h, VX-809 10 μM for 48 h, VX-770 0.1 μM for 48 h, VX-809 10 μM for 24 h followed by the addition of VX-770 0.1 μM for an additional 24 h, and a combination of VX-809 10 μM and VX-770 0.1 μM for 48 h. These treatment protocols were used in most of the methodologies described below.

### 2.4. Cell Viability

Cells were seeded in p96 plates (10,000 cells/well) and cultured under the same experimental conditions described above. Cells were treated with increasing concentrations of VX-809 and VX-770 (0, 0.01, 0.1, 1, 10 μM; four wells for each condition). Cell viability was measured using the XTT 8(2,3-Bis-2-Methoxy-4-Nitro-5-Sulfophenyl-2H-Tetrazolium) Cell Viability Kit (Cell Signaling Technology, Cat. N° 9095, Danvers, MA, USA) [62], following the manufacturer’s protocol. This assay quantifies cell viability by measuring the reduction of the yellow tetrazolium salt XTT to a soluble orange formazan catalyzed primarily by mitochondrial and cytosolic dehydrogenases [62]. The color intensity, read spectrophotometrically, is proportional to the number of metabolically active cells. Briefly, the XTT solution was prepared mixing DMEM/F12 without FBS, XTT and electron coupling solution (50:49:1). The cells were washed with PBS 1X, and the XTT solution was added. After six hours of incubation in the dark at 37 °C, the absorbance at 450 nm was measured in a Multiskan™ GO Microplate Spectrophotometer (Thermo Fisher Scientific, Waltham, MA, USA). To analyze the viability, the results were normalized to mean values corresponding to the vehicle.

### 2.5. Measurement of Mitochondrial Membrane Potential by Fluorescence Spectroscopy in a Microplate Reader

To measure the mitochondrial membrane potential (ΔΨm) by fluorescence spectroscopy [63], the fluorescent probe JC-1 was used according to the manufacturer’s instructions, with minor modifications. JC-1 is a ratiometric dye that accumulates in mitochondria depending on membrane potential (Ψm). In its monomeric form, it emits green fluorescence (~529 nm), while at high concentrations in polarized mitochondria it forms J-aggregates with red fluorescence (~590 nm) [64]. Mitochondrial depolarization is indicated by a decreased red/green fluorescence ratio. Briefly, IB3-1 cells (10,000 cells/well) were cultured in 96-well black plates (GBO Argentina S.A., Buenos Aires, Argentina; 655090). Upon reaching 70% confluence, the cells were incubated with varying concentrations of VX-809 (0; 0.01; 0.1; 10 μM) and VX-770 (0; 0.01; 0.1; 0.5; 1 μM) for 48 h. Subsequently, cells were washed three times with PBS and incubated with JC-1 (5 μg/mL) for 20 min at 37 °C. The cells were then washed three times with DMEM/F12 without FBS. The mitochondrial membrane potential was measured using a fluorescence plate reader (NOVOstar, BMG LABTECH GmbH, Ortenberg, Germany) with excitation and emission filters set to detect green fluorescence (Ex/Em = 485 ± 10/540 ± 10 nm) and red fluorescence (Ex/Em = 540 ± 10/580 ± 10 nm). The red-to-green fluorescence intensity ratio was used as an indicator of ΔΨm. Carbonyl cyanide-p-trifluoromethoxyphenylhydrazone (FCCP, mitochondrial uncoupler) treatment (20 µM) was used as a positive control of mitochondrial membrane depolarization, and its fluorescence data were subtracted to obtain the specific ΔΨm signal. The fluorescence intensity of the treatments was then normalized to control samples (vehicle).

As an alternative method to measure ΔΨm, the fluorescent probe TMRE was used as previously reported with minor modifications [65]. TMRE is a red-orange cationic fluorescent dye that accumulates in active mitochondria in response to Ψm. Loss of Ψm leads to decreased TMRE uptake and reduced fluorescence intensity [65]. TMRE has an excitation wavelength of 549 nm and an emission wavelength of 575 nm. Briefly, IB3-1 cells were cultured in 96-well black plates (GBO Argentina S.A., Buenos Aires, Argentina; 655090) with the treatments indicated above and incubated with 20 nM TMRE in DMEM/F12 medium (stock 1000× prepared as 20 µM solution in DMSO) for 30 min at 37 °C in the 5% CO_2_/air incubator. Then, cells were washed with 0.2 mL Hank’s solution (136.9 mM NaCl, 5.4 mM KCl, 1.3 mM CaCl_2_, 3.7 mM NaH_2_PO_4_, 0.4 mM KH_2_PO_4_, 4.2 mM NaHCO_3_, 0.7 mM MgSO_4_, 5.5 mM D-glucose, 10 mM HEPES) three times and the fluorescence was measured in a fluorescence plate reader (NOVOstar, BMG LABTECH GmbH, Ortenberg, Germany) at 37 °C. The filters used for TMRE were Ex/Em = 540 ± 10/590 ± 10 nm. The values corresponding to FCCP 20 µM treatment at each treatment were subtracted and plotted (to discount the background fluorescence) [66]. The fluorescence intensity of the treatments was then normalized to the vehicle control.

### 2.6. Measurement of Mitochondrial Membrane Potential by Flow Cytometry

TMRE was also used to measure ΔΨm by flow cytometry [67]. IB3-1 cells were cultured and treated as above described. Upon reaching 90% confluence, cells were incubated with 40 nM of TMRE in DMEM/F12 medium for 30 min at 37 °C in the 5% CO_2_/air incubator. Subsequently, cells were harvested by trypsin treatment and pelleted by centrifugation at 100× *g* for 10 min. The cells were then washed three times with Hank’s buffer and resuspended in the same buffer. Finally, fluorescence was measured by using a flow cytometer (BD Accuri C6, BD Bioscience, San Jose, CA, USA). FCCP treatment (20 µM) was used as a positive control of mitochondrial membrane depolarization, and the fluorescence data were subtracted to obtain the specific ΔΨm signal. The mean fluorescence intensity (MFI) of the treatments was then normalized to control values (vehicle) [66].

### 2.7. Cell-Live Imaging and Mitochondrial Morphology Analysis

Cells were seeded (1 × 10^4^ cells/cm^2^) in confocal dishes (bottom size 15 mm, Jet BioFil, Guangzhou, China) in DMEM/F12 with 5% FBS 48 h and incubated in serum-free DMEM/F12 24 h before experiments. IB3−1, S9 and C38 cells were incubated with the treatments previously described for 48 h. Mitochondria were labelled with 25 nM MitoTracker Orange CMTMRos as described in the manufacturer’s protocol. Mitochondrial images were captured in vivo by confocal microscopy (Carl Zeiss LSM510 and/or LSM980 Jena, Germany) with temperature (37 °C) and CO_2_ (5%) controlled and using a 63×/1.2 NA objective. A 543 nm laser line and BP 560−615 nm filter were used for MitoTracker. Confocal images were analyzed by using the Mitochondrial Network Analysis (MiNA) toolset plugin for Fiji (http://github.com/ScienceToolkit/MiNA) [68] and the Micro-P software version 1.1.11b_R2010b_w64 [69], as it was reported previously [26].

### 2.8. Measurement of Mitochondrial and Cellular ROS Levels by Flow Cytometry

Mitochondrial and cellular ROS levels were measured by flow cytometry as previously described by Duyndam et al. [70] with some modifications. Briefly, the cells were cultured 48 h, as previously indicated, in p100 dishes. Cellular ROS levels (cROS) were measured using the fluorescent probe DCFH-DA (Ex/Em = 488/525 nm), while mitochondrial superoxide anion levels (mtROS) were measured using the MitoSOX probe (Ex/Em = 396/590 nm). In both cases, cells were incubated in Hank’s solution containing the corresponding fluorescent probe (20 μM DCFH-DA or 2.5 μM MitoSOX) at 37 °C in the 5% CO_2_/air incubator for 40 min or 15 min, respectively. Then, cells were harvested by trypsinization and pelleted by centrifugation at 400× *g* for 5 min. The cells were washed twice with Hank’s buffer and resuspended in the same buffer. Finally, cells were resuspended in 500 μL Hank’s buffer and analyzed by flow cytometry (Accuri, BD Biosciences, FL1 for DCF and FL2 for MitoSOX). MitoTEMPO (10 µM), a mtROS scavenger [71], was used as a control to inhibit mtROS in cells exposed to VX-809. The mean fluorescence intensity (MFI) of the treatments was then normalized to control values (vehicle).

### 2.9. Mitochondrial Isolation

Mitochondria were isolated using differential centrifugation, following the protocol described by Majander et al. [25], with slight modifications as outlined in [13]. In brief, cells were cultured and treated as above described. Post-treatment, cells were kept at 0–4 °C (on ice) and washed 1× with cold PBS. They were then scraped into 1 mL of PBS and centrifuged at 600× *g* for 5 min at 4 °C. The pellet was resuspended in isolation buffer (0.25 M sucrose, 25 mM MOPS, pH 7.4) in presence of protease inhibitors: 10 mM pepstatin, 10 mM leupeptin, 100 mM PMSF, and adjusted to a concentration of 250 mg/mL. Cells were permeabilized with 0.12% *w*/*v* digitonin in isolation buffer for 30 s on ice. The sample was then diluted with three volumes of isolation buffer and centrifuged at 10,000× *g* for 20 min at 4 °C. The pellet was resuspended in 600 µL of isolation buffer and centrifuged at 800× *g* for 10 min at 4 °C. The supernatant was further centrifuged at 10,000× *g* for 20 min at 4 °C to collect the mitochondrial fraction. The mitochondrial pellet was then resuspended in 10–20 mL of MSH buffer (0.23 M mannitol, 0.07 M sucrose, 5 mM HEPES, pH 7.4). Mitochondrial protein concentration was determined using Lowry’s assay [72], after incubating mitochondrial extracts with 0.1 N NaOH for 30 min at 37 °C to solubilize mitochondrial membranes.

### 2.10. Mitochondrial NADH-Cytochrome c Reductase (mCx-I-III) Enzymatic Activity

Mitochondria were isolated from IB3-1, S9 and C38 cells as described above. The NADH-cytochrome c reductase enzymatic activity (mCx-I plus mCx-III) was spectrophotometrically measured and quantified as previously described [13,16]. Briefly, mitochondrial preparations were subjected to three freeze-thaw cycles to make them permeable to substrates. To measure the mCx-I-III activity, mitochondria (equivalent to 25 μg of proteins) were resuspended in buffer solution (100 mM H_2_KPO_4_/HK_2_PO_4_, 0.5 mM KCN, 200 μM NADH, 100 μM oxidized cytochrome c, at pH 7.4). The reduction of cytochrome c was recorded by monitoring the increase in absorbance at 550 nm per minute for 4 min at 30 °C. Measurements were carried out in a Multiskan™ GO Microplate Spectrophotometer (Thermo Fisher Scientific, Waltham, MA, USA). Results were nmol cytochrome c reduced/min/mg protein.

### 2.11. ATP Basal Levels

Cells were grown for 24 h on 96-well black plates and treated as previously described. Basal ATP levels were determined in cells by the chemiluminescent detection assay using the luciferine-luciferase reaction. A calibration curve using ATP as standard (0–10 nmoles) was carried out [73]. Cells were permeabilized with 0.01% digitonin, and ATP basal content was immediately measured in a reaction medium containing Hank’s buffer at pH 7.4, 40 μM luciferine, 1 μg/mL luciferase at 30 °C. Oligomycin (0.01 mg/mL) and FCCP (20 μM) were used as a negative control. ATP levels were measured by luminescence using a NOVOstar microplate reader (BMG LABTECH GmbH, Ortenberg, Germany). Protein concentration was measured using the micro-BCA protein Assay Kit (Thermo Fisher Scientific, REF 23235, Rockford, IL, USA) in each well. ATP levels were expressed as nmol ATP/mg protein.

### 2.12. Measurement of Relative Intracellular Chloride

First, the intracellular Stern–Volmer constant for MQAE quenching by Cl^−^ (K_Cl_^−^) was determined in IB3-1, S9 and C38 cells using the double ionophore method with modifications as previously described [74,75]. Briefly, cells were grown for 24 h on 96-well black plates (Greiner Bio-One, Germany, Cat. N° 655090), washed twice with PBS 1X, and incubated with 5 mM MQAE in serum-free DMEM/F12 for 40 min at 37 °C [76,77]. A Cl^−^ calibration curve was performed using two high K buffers: (a) High KCl buffer: 1.3 mM Ca-gluconate, 140 mM KCl, 3.7 mM NaH_2_PO_4_, 4.2 mM NaHCO_3_, 0.7 mM MgSO_4_, 5.5 mM D-glucose, 10 mM HEPES and (b) High KNO_3_ buffer: 1.3 mM Ca-gluconate, 140 mM KNO_3_, 3.7 mM NaH_2_PO_4_, 4.2 mM NaHCO_3_, 0.7 mM MgSO_4_, 5.5 mM D-glucose, 10 mM HEPES). The ionophores nigericin (5 µM) and tributyltin (10 µM) were included in each concentration point. MQAE fluorescence intensity was measured at 37 °C using a NOVOstar fluorescence microplate reader (BMG LABTECH GmbH, Ortenberg, Germany) with excitation/emission wavelength of 350 ± 10 nm and 460 ± 10 nm, respectively. For each Cl^−^ concentration, eight wells were analyzed in three independent experiments. To ensure equilibrium between the intracellular and extracellular Cl^−^ concentrations, cells were incubated 10 min prior to measurement. The average fluorescence value for each Cl^−^ concentration was then used to construct the Stern–Volmer plot. Once MQAE’s sensitivity to intracellular Cl^−^ changes was confirmed, cells were seeded under the same conditions and treated with CFTR modulators as previously described. Following treatments, cells were washed three times with PBS 1X, loaded with MQAE as described above, and incubated in Hank’s buffer for 5 min at 37 °C before measurement. The fluorescence intensity of the treated cells was normalized to vehicle control to determine the relative intracellular Cl^−^ concentration.

### 2.13. Protein Extraction and Western Blot Analysis (WBs) 

Cells were seeded (4 × 10^5^ cells/cm^2^) in p60 plates in the same conditions and treatments with CFTR modulators described above. Cells were washed twice with cold PBS, scraped with cold extraction buffer (10 mM Tris, 100 mM NaCl, 0.1% SDS, 0.5% sodium deoxycholate, 1% Triton X-100, 10% glycerol, pH 7.4) containing the protease inhibitor cocktail plus phosphatase inhibitors, incubated 1 h on ice and centrifuged at 14,000× *g* for 20 min at 4 °C. The supernatants were used to quantify the protein concentration by Lowry, and the rest of the sample was incubated in cracking buffer at 95 °C for 5 min and stored at −20 °C until use. Total protein extracts (50 μg) were separated on a 10% SDS-PAGE and transferred to nitrocellulose membranes. Membranes were blocked with 5% BSA plus 0.05% Tween-20 in TBS and then incubated with primary antibodies: anti-Actin, anti-DRP1 and anti-mitofusin-1, dilutions 1:1000 in 5% BSA plus 0.05% Tween-20 in TBS overnight at 4 °C. Membranes were washed three times with PBS plus Tween-20 (0.1% *v*/*v*) for 10 min and incubated for 1 h with goat IgG anti-mouse or anti-rabbit antibody coupled to HRP (dilution 1:2000 in TBS plus Tween-20, 0.05% *v*/*v*), washed three times with TBS plus Tween-20 (0.1% *v*/*v*) for 10 min. Finally, membranes were incubated with 2.5 mL of solution A (25 µL of luminol 250 mM, 11 µL of p-coumaric acid 90 mM, 250 µL Tris 1 M pH 8.8 and 2.22 mL H_2_O) and 2.5 mL of solution B (4.55 µL H_2_O_2_ 30%, 250 µL Tris 1 M pH 8.8 and 2.25 mL H_2_O). Results were visualized by chemiluminescence using an ImageQuant LAS 4000 system (GE Healthcare Life Sciences, Piscataway, NJ, USA) or a ChemiDoc MP system (Bio-Rad Laboratories, Hercules, CA, USA). The expression was quantified by densitometry using ImageJ software (1.54f version).

### 2.14. Iodide Efflux Assay

CFTR activity was estimated by an iodide efflux assay using the chloride-sensitive fluorescent dye MQAE, and measured by flow cytometry, as previously described with some modifications [75,78,79,80]. Four buffer solutions were prepared: (a) iodide buffer (135 mM NaI, 10 mM D-glucose, 1 mM CaSO_4_, 1 mM MgSO_4_, 10 mM HEPES, 2.4 mM K_2_HPO_4_, 0.6 mM KH_2_PO_4_), (b) nitrate buffer (135 mM NaNO_3_, 10 mM D-glucose, 1 mM CaSO_4_, 1 mM MgSO_4_, 10 mM HEPES, 2.4 mM K_2_HPO_4_, 0.6 mM KH_2_PO_4_), (c) CFTR activator buffer (nitrate buffer supplemented with 200 µM dibutyryl cAMP, 200 µM IBMX, and 20 µM isoproterenol), and (d) quenching solution (iodide buffer containing 5 µM valinomycin). Briefly, IB3-1 and S9 cells were seeded in 24-well plates (25,000 cells/cm^2^) and cultured under the same experimental conditions described above. Treated and untreated cells were incubated with 5 mM MQAE in the iodide buffer (a) for 40 min at 37 °C. Then, cells were washed with that same buffer three times, harvested by trypsin treatment. Trypsin was inactivated with 5% FBS prepared in the same buffer assigned to each subsequent measurement condition. Cells were pelleted by centrifugation at 100× *g* for 10 min and resuspended in 200 µL of the corresponding buffer to measure the MQAE signal. Fluorescence was collected by using a flow cytometer (Accuri, BD Bioscience). *F*/*F_i_* − 1 was calculated, where *F_i_* represents the fluorescence of the cells treated with the quenching solution, and *F* is the fluorescence for each incubation buffer. Finally, the Δ*F*/*F_i_* − 1 was calculated by subtracting the *F*/*F_i_* − 1 value obtained in cells incubated with nitrate buffer (basal condition) from those obtained in cells stimulated with the CFTR activator solution. The difference reflects the relative CFTR-dependent response. Values were then normalized to vehicle controls, and fold change was calculated accordingly.

### 2.15. Statistics

The results from confocal microscopy correspond to images randomly selected from independent experiments (n = 5). At least 10 images were acquired for each treatment and 10–30 cells per experiment. Bars and dots analysis shows the mean ± SEM of at least 3 independent experiments normalized to control. WBs results corresponded to the average of at least 3 independent experiments (mean ± SEM). One-way ANOVA and Tukey’s or Paired-Student’s t-test were applied to determine significant differences (GraphPad Prism Software 10.0), as indicated in figure legends (* *p* < 0.05, ** *p* < 0.01, and *** *p* < 0.001).

## 3. Results

### 3.1. Effects of CFTR Modulators on Cell Viability and Mitochondrial Membrane Potential

**Figure 1:**
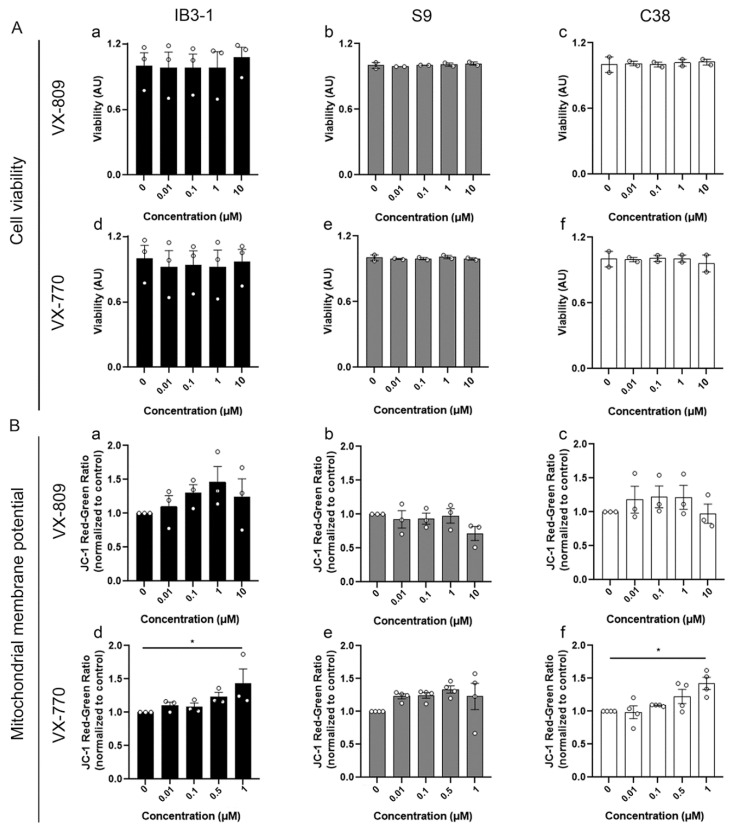
VX-809 and VX-770 at different concentrations do not affect mitochondrial cell viability or membrane potential. (**A**) Bars and dots (replicates) represent cell viability, measured by XTT assay, upon treatment with increasing concentrations of VX-809 (**a**–**c**) or VX-770 (**d**–**f**). (**B**) Bars and dots (replicates) show the JC-1 Red–Green fluorescence ratio, normalized to control, after treatment with VX-809 (**a**–**c**) or VX-770 (**d**–**f**). Measurements were obtained using a fluorescence plate reader. All measurements were performed four times, and data are expressed as mean ± SEM from at least three independent experiments (n = 3 or n = 4). Circles represent individual data points. * *p* < 0.05 indicates a statistically significant difference compared to the control group. Statistical analysis was performed using one-way ANOVA.

Initially, we evaluated the potential effects of individual treatments with the CFTR modulators on cell viability and mitochondrial membrane potential (ΔΨm) to assess the impact of VX-809 and VX-770 on overall cellular health. Cell viability was measured using XTT assays in IB3-1 (CF cells), S9 (wild type-CFTR) and C38 cells (expressing functional but truncated CFTR), a panel of related bronchial epithelial cell lines with distinct CFTR expression profiles. IB3-1 cells (ΔF508/W1282X [57]) served as a CF model with limited endogenous CFTR activity. To assess whether the effect of CFTR modulators depends on CFTR expression and function, we included S9 cells, which ectopically express WT-CFTR and serve as a reference condition to assess pharmacological correction in IB3-1 cells. Additionally, the C38 cell line, which expresses a truncated yet functional CFTR variant with high basal activity [61], was included to explore any potential involvement of the CFTR C-terminal region in modulator responses. This approach enabled us to distinguish the respective contribution of both CFTR expression level and structural integrity to the observed mitochondrial effects. Cells were treated with various concentrations of each modulator for 48 h. The results showed no significant changes in cell viability (Figure 1A, panels a–f), suggesting that the concentrations used were non-cytotoxic.

To further investigate the individual effects on mitochondrial function, ΔΨm was measured using JC-1 probe, which measures the red/green fluorescence ratio, under the same conditions as the viability analysis. As shown in Figure 1B, treatment with VX-809 did not significantly alter the ΔΨm in either CF or corrected-CF cells (Figure 1B, panels a–c). In contrast, treatment with VX-770 at a concentration of 1 µM led to a significant increase in ΔΨm in both CF IB3-1 and C38 cells (Figure 1B, panels d and f), suggesting a nonspecific effect at this concentration, independent of CFTR activity.

Given the potential off-target effects on ΔΨm, we decided to use a concentration of 0.1 µM of VX-770 for the following experiments.

### 3.2. Co-Treatment with VX-809 and VX-770 and Mitochondrial Fragmentation

To determine whether the CFTR modulator co-treatment affects mitochondrial dynamics, IB3-1 (CF) cells were treated with VX-809 (10 μM) and VX-770 (0.1 μM), or vehicle (DMSO) as a control. Cells in one treatment group were incubated with VX-809 for 24 h, then VX-770 was added, and incubation continued for another 24 h (hereafter referred to as VX-809 48 h + VX-770 24 h). This strategy aimed to promote CFTR correction at the plasma membrane prior to potentiation with VX-770. In a second group, cells were treated with both VX-809 and VX-770 simultaneously for 48 h (hereafter referred to as VX-809 48 h + VX-770 48 h), as it was established in the treatments for CF patients [56,81].

**Figure 2:**
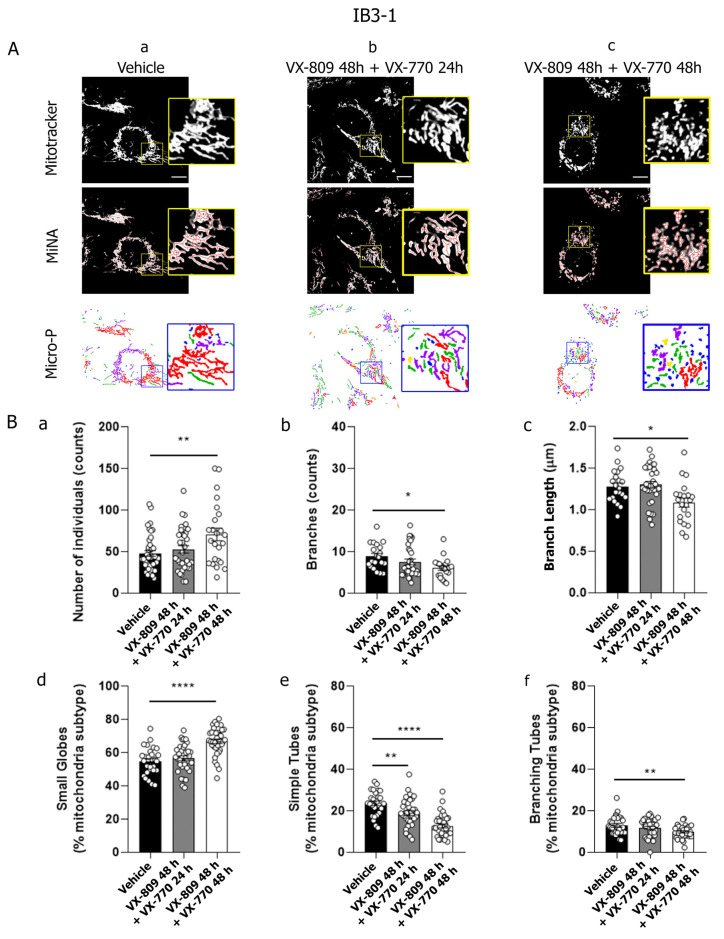
The combination of VX-809 and VX-770 increased mitochondrial fragmentation morphology in IB3-1 cells. (**A**) (**a**–**c**) Representative images observed through confocal microscopy of IB3-1 cells labeled with the fluorescent probe MitoTracker Orange and treated with VX-809 (10 µM) combined with VX-770 (0.1 µM). Mitochondrial morphology was analyzed using the MiNA and Micro-P tools. Color code for Micro-P mitochondria classification: small globular mitochondria (blue), simple tube (green, unbranched mitochondria), and branching tubes (purple, high connectivity). The images were randomly selected with a n = 5 from independent experiments. Scale bar: 10 µm. (**B**) (**a**–**c**) Bars and dots (33 replicates) show the quantification of individual mitochondrial structures, mitochondrial network length, and the number of mitochondrial networks analyzed by MiNA. (**d**–**f**) Bars and dots (33 replicates) show the quantification of small globes, simple tubes, and branching tubes analyzed by Micro-P. Data were analyzed by ANOVA one-way and Tukey post hoc test. * *p* < 0.05, ** *p* < 0.01, **** *p* < 0.0001 indicate significant differences compared to the control group (DMSO).

After the treatments, mitochondria were labeled with the MitoTracker Orange probe, and mitochondrial morphology was analyzed in live cells using confocal microscopy. MiNA analysis showed that the treatment with VX-809 48 h + VX-770 24 h did not result in significant differences in mitochondrial morphology compared to control cells (DMSO). In contrast, combined treatment with VX-809 and VX-770 for 48 h led to increased mitochondrial fragmentation parameters in IB3-1 cells (Figure 2B, panels a–c), including an increased number of individual mitochondrial structures (Figure 2B, panel a), along with a significant decrease in both the number of branches (Figure 2B, panel b) and the mitochondrial network length (branch length) (Figure 2B, panel c). In addition, analysis using Micro-P software indicated a significant increase in the small globe mitochondrial subtype and a decrease in simple tube and branching tubes mitochondrial subtypes (Figure 2B, panels d–f). Together, these results suggest a shift toward smaller, less interconnected mitochondria in IB3-1 cells treated with VX-809 and VX-770 for 48 h.

**Figure 3:**
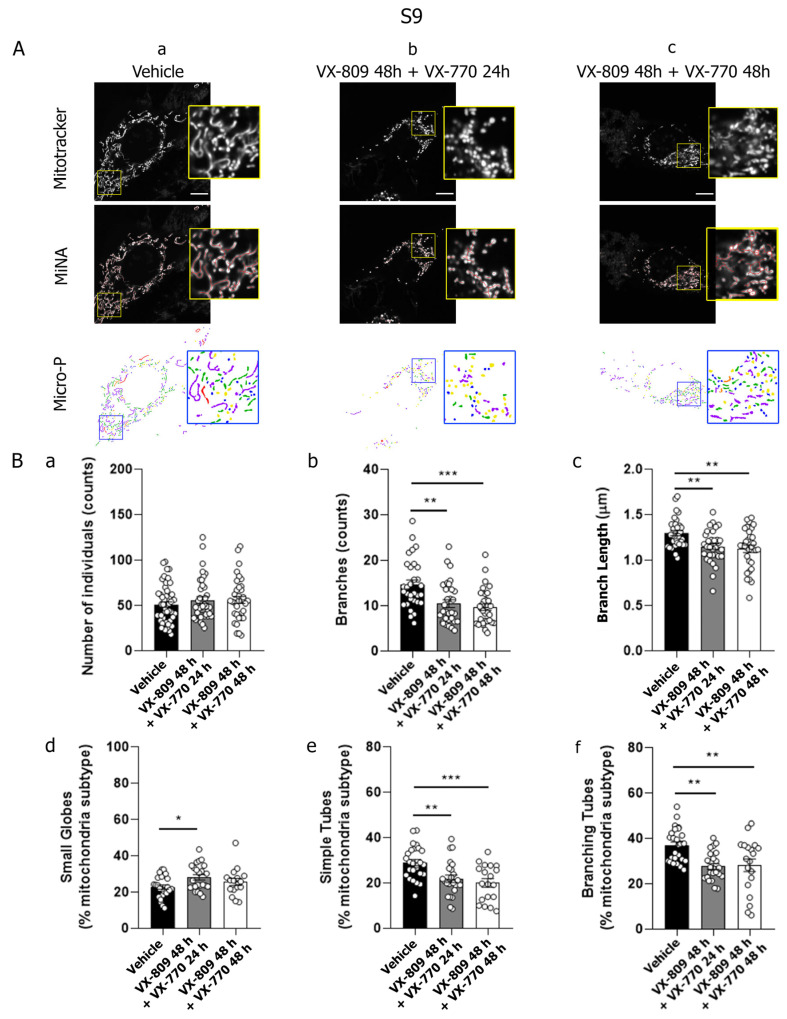
The combination of VX-809 and VX-770 increased mitochondrial fragmentation morphology in S9 cells. (**A**) (**a**–**c**) Representative images observed through confocal microscopy of S9 cells labeled with the fluorescent probe MitoTracker Orange and treated with VX-809 (10 µM) combined with VX-770 (0.1 µM). Mitochondrial morphology was analyzed using the MiNA and Micro-P tools. Color code for Micro-P mitochondria classification: small globular mitochondria (blue), simple tube (green, unbranched mitochondria), and branching tubes (purple, high connectivity). The images were randomly selected with a n = 5 from independent experiments. Scale bar: 10 µm. (**B**) (**a**–**c**) Bars and dots (32 replicates) show the quantification of individual mitochondrial structures, mitochondrial network length, and the number of mitochondrial networks analyzed by MiNA. (**d**–**f**) Bars and dots (32 replicates) show the quantification of small globes, simple tubes, and branching tubes analyzed by Micro-P. Data were analyzed by one-way ANOVA and Tukey post hoc test. * *p* < 0.05, ** *p* < 0.01, *** *p* < 0.001, indicate significant differences compared to the control group (DMSO).

**Figure 4:**
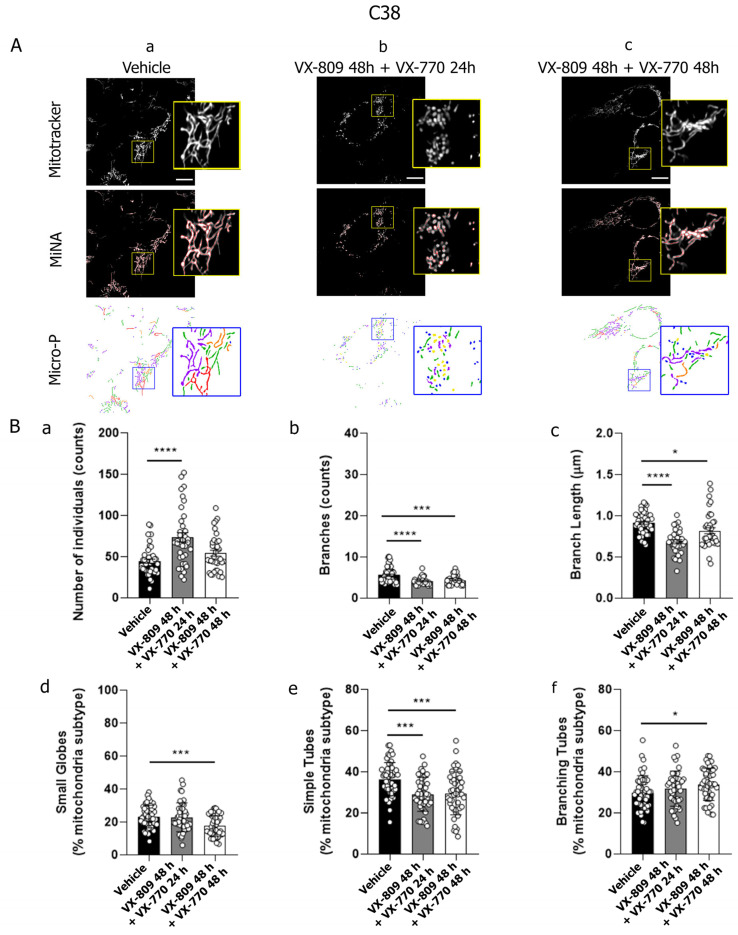
The combination of VX-809 and VX-770 increased mitochondrial fragmentation morphology in C38 cells. (**A**) (**a**–**c**) Representative images observed through confocal microscopy of C38 cells labeled with the fluorescent probe MitoTracker Orange and treated with VX-809 (10 µM) combined with VX-770 (0.1 µM). Mitochondrial morphology was analyzed using the MiNA and Micro-P tools. Color code for Micro-P mitochondria classification: small globular mitochondria (blue), simple tube (green, unbranched mitochondria), and branching tubes (purple, high connectivity). The images were randomly selected with a n = 5 from independent experiments. Scale bar: 10 µm. (**B**) (**a**–**c**) Bars and dots (37 replicates) show the quantification of individual mitochondrial structures, mitochondrial network length, and the number of mitochondrial networks analyzed by MiNA. (**d**–**f**) Bars and dots (37 replicates) show the quantification of small globes, simple tubes, and branching tubes analyzed by Micro-P. Data were analyzed by one-way ANOVA and Tukey post hoc test. * *p* < 0.05, *** *p* < 0.001, **** *p* < 0.0001 indicate significant differences compared to the control group (DMSO).

Similar results were observed in S9 cells following treatment with VX-809 and VX-770 for 48 h (Figure 3). However, unlike IB3-1 cells, the sequential treatment with VX-809 followed by the addition of VX-770 (VX-809 48 h + VX-770 24 h) also induced changes that suggest increased mitochondrial fission. Specifically, MiNA analyses showed a significant decrease in both the number of branches and their length compared to control cells (Figure 3B, panels b and c). In addition, Micro-P analysis showed a significant increase in the population of small globe mitochondria (Figure 3B, panel d), along with a significant decrease in both simple tube and branching tubular mitochondria (Figure 3B, panels e and f). These results agreed with those obtained in C38 cells by using MiNA analysis (Figure 4B, panels a–c). However, Micro-P analysis in C38 cells showed a significant decrease in both small globes and simple tubes mitochondrial subtypes (Figure 4B, panels d and e), accompanied by a significant increase in the branching tubular subtype (Figure 4B, panel f).

**Figure 5:**
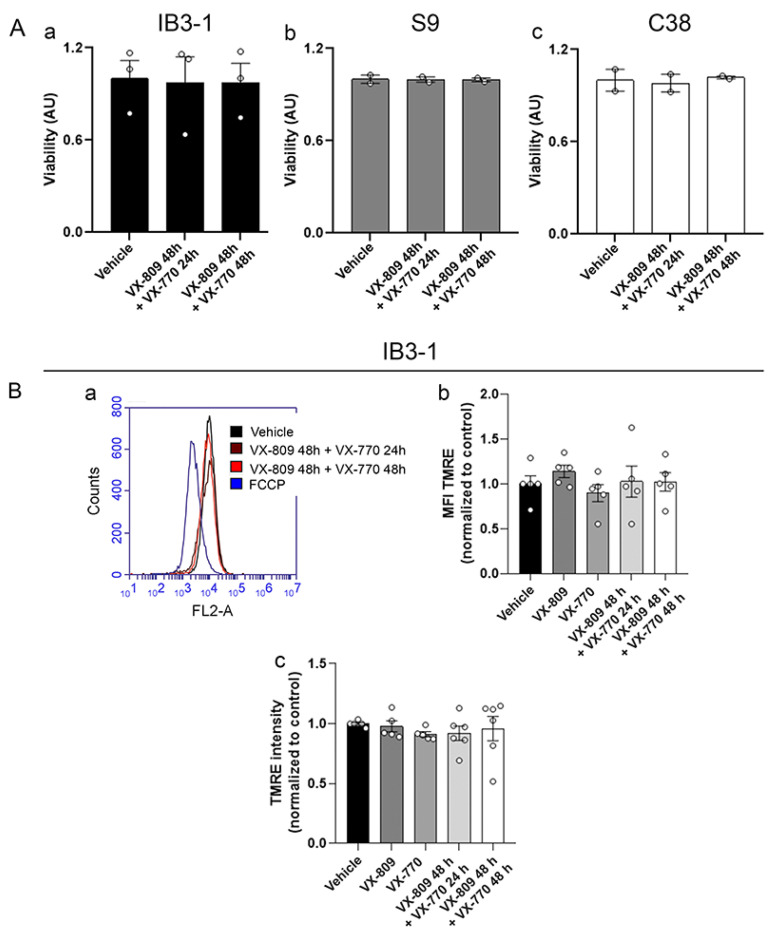
Different treatments do not affect mitochondrial membrane potential. (**A**) (**a**–**c**) Bars and dots (replicates) showing the quantification of cell viability when treated with a combination of VX-809 10 µM and VX-770 0.1 µM. (**B**) (**a**) Flow cytometry histogram showing the TMRE fluorescence of cells treated with DMSO (vehicle), VX-809 (10 µM) for 24 h followed by the addition of VX-770 (0.1 µM) for an additional 24 h, VX-809 (10 µM) and VX-770 (0.1 µM) 48 h, and with FCCP (20 µM) as a positive control for mitochondrial depolarization. (**b**) Bars and dots (replicates, n = 5) show the quantification of the TMRE fluorescence measured by flow cytometry normalized to control at individual and combined treatments with VX-809 and VX-770. (**c**) Bars and dots (replicates, n = 6) show the quantification of the TMRE fluorescence measured by microplate reader normalized to control at individual and combined treatments with VX-809 and VX-770. Measurements were performed in quadruplicate, and data are expressed as mean ± SEM. Means were compared using one-way ANOVA.

It was then studied whether the changes in mitochondrial morphology induced by VX-809 and VX-770 treatments were associated with alterations in cell viability or the mitochondrial membrane potential (ΔΨm). Cell viability was measured by using XTT assays; IB3-1, S9 and C38 cells were treated with DMSO or VX-809 and VX-770 together, as described above. No significant changes were observed in cell viability, suggesting that the treatments used were non-cytotoxic at those concentrations (Figure 5A, panels a–c). On the other hand, ΔΨm was measured using the TMRE probe through flow cytometry (Figure 5B, panels a and b) and spectrofluorometry (Figure 5B, panel c) in IB3-1 cells. The mitochondrial uncoupler FCCP (20 µM) was used as a positive control for depolarization. No significant changes in ΔΨm were observed between cells treated with CFTR modulators and control cells (vehicle).

### 3.3. Effects of Lumacaftor (VX-809) or Ivacaftor (VX-809) Alone on Mitochondrial Morphology and ROS Production

After observing that the combination of the two drugs causes mitochondrial fragmentation, we investigated the independent effects of the potentiator (VX-770, 0.1 µM) and the corrector (VX-809, 10 µM) on mitochondrial morphology and ROS production in CF IB3-1 cells. Considering that CFTR modulators induced comparable mitochondrial alterations across CFTR-rescued (S9 and C38) and CF (IB3-1) cell lines, we continued our analysis using IB3-1 cells as a representative CF model. This strategy allowed us to specifically examine the mitochondrial consequences of CFTR modulation in a CF context, where CFTR function is inherently compromised. The cells were analyzed using MiNA or Micro-P software. No significant changes in mitochondrial morphology parameters were detected with either VX-770 or VX-809 isolated treatment (Figure S1).

**Figure 6:**
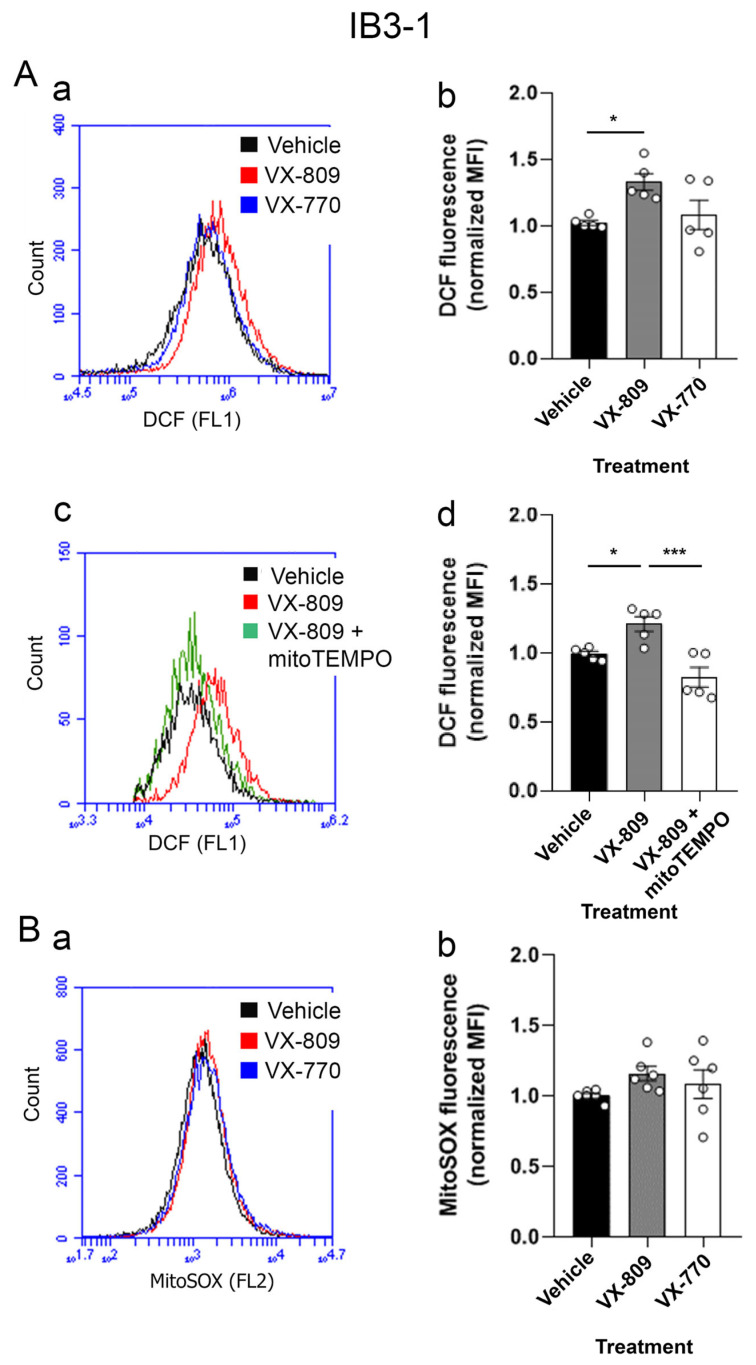
VX-809 induced cellular ROS levels in IB3-1 cells. (**A**) (**a**,**b**) Quantification of cellular ROS levels measured by flow cytometry in cells treated with CFTR modulators. (**c**,**d**) Quantification of cellular ROS levels measured by flow cytometry in cells treated with VX-809 with and without MitoTEMPO (10 µM). (**B**) (**a**,**b**) Quantification of mitochondrial ROS levels in treated groups compared to the control group. Measurements were performed in triplicate, and data are expressed as mean ± SE from five independent experiments (n = 5). * *p* < 0.05, *** *p* < 0.001 indicate significant differences compared to control.

On the other hand, cellular ROS (cROS) and mitochondrial ROS (mtROS) levels have been widely reported as elevated in different CF models [12,13,15,19]. To evaluate whether individual treatment with the corrector VX-809 (which theoretically increases CFTR levels at the plasma membrane) or the potentiator VX-770 (which enhances the channel’s open probability) [82] could reduce ROS production, we measured cROS and mtROS in CF cells using DCFH-DA and MitoSOX fluorescent probes, respectively. Notably, the cROS levels were significantly increased in IB3-1 cells treated with the corrector VX-809 (1.331 ± 0.064, n = 3), while the potentiator VX-770 showed no effect (1.083 ± 0.111, n = 3) compared to control (1.023 ± 0.019, n = 3) (Figure 6A, panels a and b). Although the mtROS did not show significant changes (Figure 6B, panels a and b), co-treatment with the mitochondrial antioxidant MitoTEMPO (10 µM) effectively blocked the VX-809-induced increase in cROS (0.679 ± 0.072, n = 3) (Figure 6A, panels c and d), suggesting that mitochondria are the primary source of the cROS elevation observed with VX-809 treatment.

The antioxidant treatment with MitoTEMPO (10 µM) and NAC (5 mM) did not appear to prevent the mitochondrial fragmentation induced by the combined treatment with VX-770 and VX-809 (Supplementary Figure S2), suggesting that the increased ROS levels caused by VX-809 might not be the main contributor to the observed alterations in mitochondrial morphology.

### 3.4. Effects of Co-Treatment with VX-809 and VX-770 for 48 h on NADH Cytochrome c Reductase Activity and ATP Content

**Figure 7:**
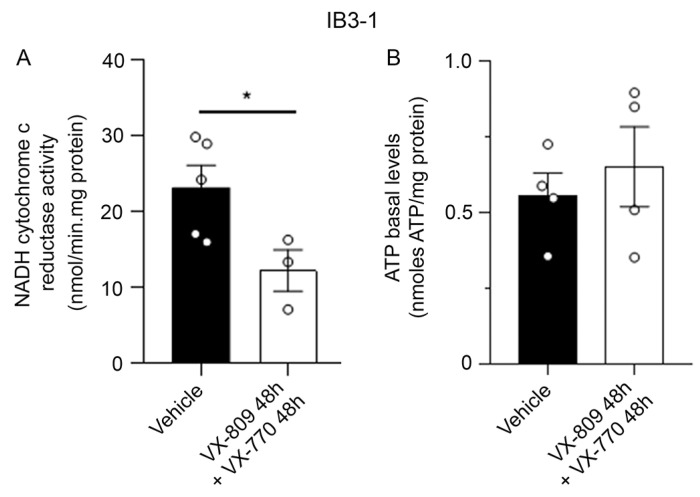
Co-treatment with VX-809 and VX-770 for 48 h decreases NADH cytochrome c reductase activity but maintains ATP basal content. (**A**) NADH cytochrome c reductase activity. Bars and dots (replicates) show the quantification of mCx-I-III activity. Measurements were performed in triplicate, and data are expressed as mean ± SE from three independent experiments (n = 3). * *p* < 0.05 indicates significant differences compared to the control group (DMSO). Means were compared using Student’s T test. (**B**) ATP basal content. Bars and dots (replicates) show the quantification of ATP basal level. Measurements were performed in triplicate, and data are expressed as mean ± SE from three independent experiments (n = 4). Means were compared using Student’s T test.

To investigate the potential impact of VX-809 and VX-770 treatment on mitochondrial function, NADH-cytochrome c reductase enzymatic activity (mCx-I coupled to mCx-III) and ATP basal content were determined. IB3-1 cells were treated with the following conditions: VX-809 and VX-770 in combination for 48 h and vehicle (DMSO) as a control. Mitochondrial complex I enzymatic activity was evaluated spectrophotometrically, while ATP basal content was determined by a luciferin-luciferase luminescence assay. Co-treatment with VX-809 and VX-770 for 48 h resulted in a significant decrease in mCx-I-III activity (12.21 ± 2.71 mol/min.mg protein) compared with the control condition (23.17 ± 2.90 nmol/min.mg protein, n = 3, *p* < 0.05) (Figure 7A). On the other hand, ATP basal content remained unchanged after co-treatment with VX-809 and VX-770 for 48 h (0.65 ± 0.13 nmol ATP/mg protein, n = 4) compared to control (0.56 ± 0.08 nmol ATP/mg protein) (Figure 7B).

### 3.5. Expression of Canonical Mitochondrial Dynamic Proteins MFN1 and DRP1 Does Not Appear to Explain Mitochondrial Fragmentation in IB3-1 and S9 Cells Treated with VX-809 and VX-770

**Figure 8:**
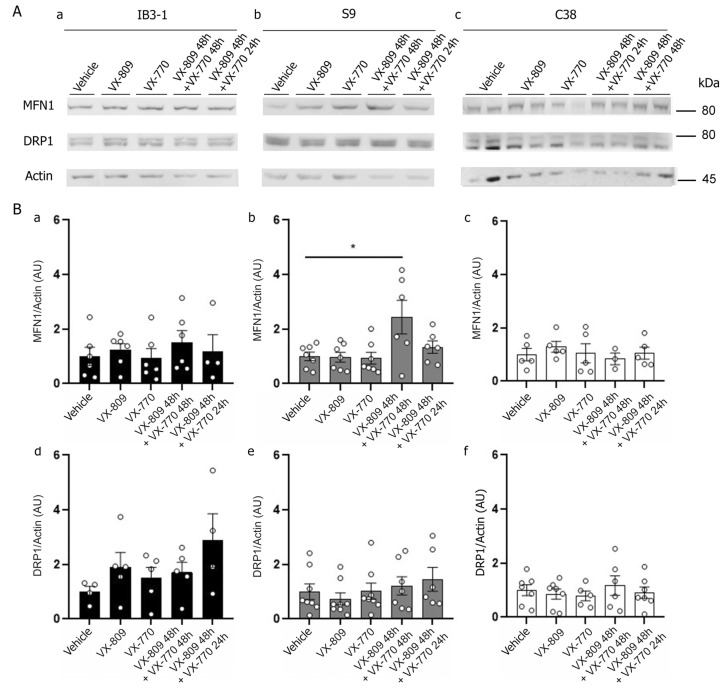
Expression of mitochondrial dynamics proteins DRP1 and MFN1 in cells treated with VX-809 and VX-770. (**A**) (**a**–**c**) Representative WBs corresponding to the levels of MFN1 and DRP1 (proteins involved in mitochondrial fission/fusion balance regulation) and actin expressed in IB3-1 (**a**), S9 (**b**), and C38 (**c**) cells. (**B**) (**a**–**c**) Ratio of MFN1/actin. (**d**–**f**) Ratio of DRP1/actin. Densitometric analysis was carried out using ImageJ software, and data were graphed as a % relative to the control group. Data were expressed as mean ± SE (n = 5–7 independent experiments). The open circles in bar graphs represent the mean values of each independent experiment, and the bars represent the average of the mean values. Two-way ANOVA for the effect of genotype; * *p* ≤ 0.05, indicate significant differences compared to the control group.

To further analyze the changes in mitochondrial morphology, the expression of proteins involved in mitochondrial dynamics, MFN1 and DRP1, was measured by Western Blot (Figure 8A) in IB3-1, S9 and C38 cells treated with CFTR modulators individually or in combination at the indicated times. Two of the main proteins involved in the mitochondrial dynamics canonical pathway are MFN1, located in the outer mitochondrial membrane, which mediates mitochondrial fusion, whereas DRP1, a cytoplasmatic protein, is a key regulator of mitochondrial fusion. Protein levels were quantified as MFN1/Actin (Figure 8B) and DRP1/Actin (Figure 8B, panels c,d) ratios.

In IB3-1 cells, treatment with both modulators (VX-809 for 48 h and VX-770) resulted in a non-significant trend toward increased expression of both DRP1 and MFN1. In contrast, S9 cells showed an increase in MFN1 expression after 48 h of combined treatment, while no changes were observed in DRP1 levels. Taken together, these results suggest that the expression levels of MFN1 and DRP1 alone do not account for the mitochondrial fragmentation observed under these conditions.

### 3.6. Intracellular Chloride Concentration and Iodide Efflux in CF IB3-1 Cells Treated with VX-809 and VX-770

**Figure 9:**
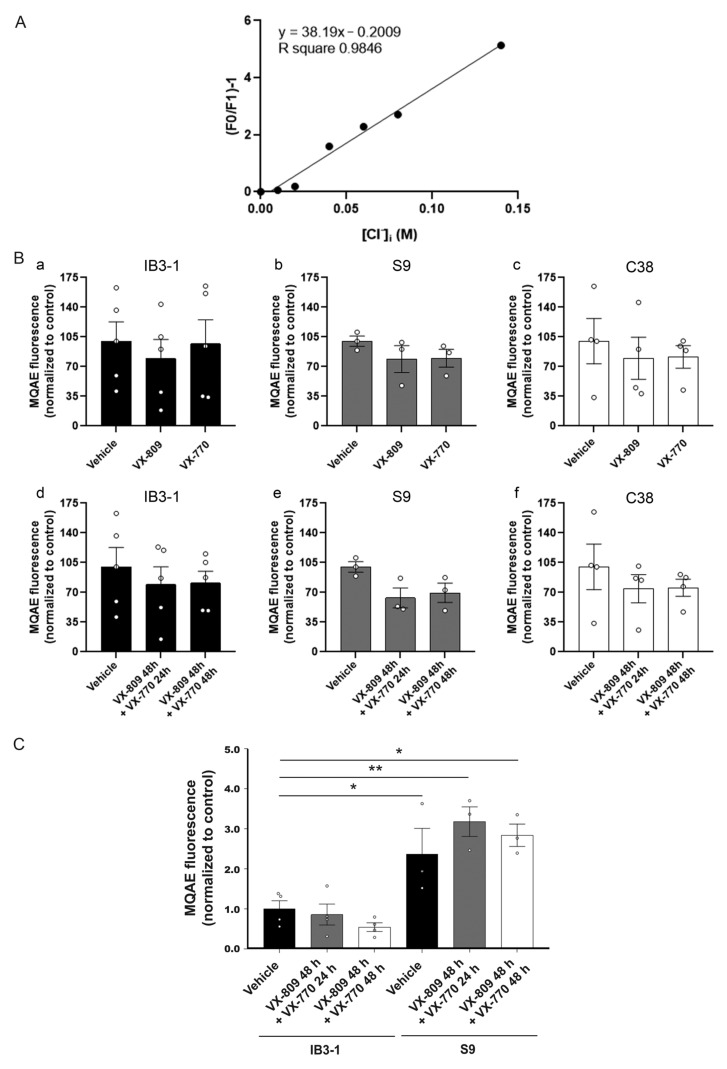
Relative intracellular chloride concentration and CFTR activity in cells treated with VX-809 and VX-770. (**A**) Stern–Volmer constant for MQAE. (**B**) Bars and dots (replicates) show the quantification of MQAE fluorescence normalized to control in IB3-1 (**a**,**d**), S9 (**b**,**e**) and C38 (**c**,**f**) cells. (**C**) Bars and individual data points represent iodide efflux quantification measured using MQAE and analyzed by flow cytometry. Measurements were performed in triplicate, and data are expressed as mean ± SE from three independent experiments (n = 3). Means were compared using one-way ANOVA, * *p* ≤ 0.05, ** *p* ≤ 0.01, indicate significant differences compared to the control group.

To determine whether treatment with the CF modulators influences intracellular chloride concentration [Cl^−^]_i_, cells cultured in 96-wells plates were incubated with the MQAE probe for 1 h, and fluorescence was then measured using a fluorescence plate reader. MQAE is a membrane-permeable fluorescent Cl^−^ indicator that is highly sensitive to Cl^−^ and is rapidly quenched by Cl^−^ [83]. The intracellular Stern–Volmer constant for MQAE was 38 M^−1^, with similar results to those previously reported [84] (Figure 9A). However, no significant changes in [Cl^−^]_i_ were observed following treatment with CF modulators in any of the cell lines (Figure 9B, panels a–f), suggesting that mitochondrial fragmentation induced by CFTR modulators was independent of changes in the [Cl^−^]_i_.

To analyze whether treatment with VX-809/VX-770 induced CFTR activity, iodide efflux was assessed in IB3-1 cells (Figure 9C). S9 cells expressing WT-CFTR were included as a control. As shown in Figure 9C, CFTR stimulation with cAMP did not result in significant changes in iodide efflux among treatments, suggesting that CFTR activity was not improved by the combined modulators. Nevertheless, iodide efflux showed a non-significant trend toward an increase in S9 cells treated with the modulators, while a clear and significant increase was observed in untreated S9 control cells compared to the CF ones, as expected.

## 4. Discussion

In this work, we investigated changes in mitochondrial morphology and analyzed key parameters of mitochondrial function in response to the CFTR modulators lumacaftor (VX-809) and ivacaftor (VX-770) in heterozygous CF IB3-1 cells [55,56]. The combined use of VX-809 and VX-770 constitutes the first approved targeted therapy for CF patients homozygous for the ΔF508 mutation, showing improvements in lung function and attenuating inflammatory responses in CF airway epithelial cells [85,86]. However, the limited efficacy of this therapy in CF patients carrying heterozygous ΔF508 mutations remains poorly understood.

First, we ruled out cytotoxic effects of the individual treatments (VX-770 or VX-809) at different concentrations or in combination for 48 h in the different cell lines used. However, an increase in mitochondrial membrane potential (ΔΨm) was observed with 1 µM of VX-770 in IB3-1 CF cells and C38 cells, prompting us to use 0.1 µM to minimize potential off-target effects that might influence mitochondrial morphology. This agrees with previous reports describing adverse effects of VX-770 at higher concentrations [87,88]. Indeed, 10 µM VX-770 can impair VX-809-mediated rescue of ΔF508-CFTR [88,89,90] and destabilize other membrane solute carriers, such as SLC26A3, SLC26A9, and SLC6A14 [91], probably due to nonspecific effects of VX-770 and its derivatives on the lipid bilayer, attributed to their predicted lipophilicity [91]. Such properties could also explain the observed increase in ΔΨm at high ivacaftor concentrations. Recently, Guimbellot et al. [92] reported that intracellular VX-770 levels can exceed plasma concentrations and accumulate within patient cells. Altogether, the evidence that elevated concentrations of VX-770 may hinder CFTR rescue supports our choice of using 0.1 µM VX-770 in subsequent experiments.

Mitochondrial network morphology was analyzed in IB3-1 CF cells and compared with control cell lines S9 and C38 (cells stably expressing functional CFTR) by quantitatively assessing parameters obtained from the MiNA plugin and Micro-P software. Data showed that combined treatment with VX-809 (10 μM) and VX-770 (0.1 μM) induced mitochondrial fragmentation in both CF and control cell lines. However, a key difference was observed in S9 and C38 cells, showing mitochondrial fragmentation when treated with VX-809 for 24 h followed by the addition of VX-770 for another 24 h, whereas CF cells showed this pattern when cells were incubated for 48 h with both modulators. This suggests that functional CFTR may accelerate the response to VX-770, supporting the hypothesis that CFTR activity modulates mitochondrial dynamics. This aligns with previous work by Rimessi et al. [93], who demonstrated that *Pseudomonas aeruginosa* infection altered mitochondrial morphology in CF cells but not in CFTR-expressing cells. In their study, infection induced intracellular calcium dysregulation, pro-inflammatory cytokine production, mitochondrial membrane depolarization, and increased ROS levels [93]. In contrast, our data show that mitochondrial fragmentation induced by CFTR modulators was not accompanied by ΔΨm changes or reduced cell viability, suggesting a non-apoptotic mechanism [94,95]. Our previous findings support this notion, as CFTR dysfunction alone was sufficient to induce mitochondrial fragmentation [26], reinforcing CFTR’s role as a signaling hub in mitochondrial homeostasis during external stress. These observations agree with Hamilton et al. [96], who reported increased mitochondrial fragmentation in CF macrophages infected with *Burkholderia cenocepacia*.

Individual treatments with VX-809 or VX-770 did not induce significant changes in mitochondrial morphology in any of the three cell lines analyzed, suggesting that both corrector and potentiator were required to trigger mitochondrial fragmentation (Figure S1). Interestingly, VX-809 alone increased cellular ROS, an effect that was abolished by co-treatment with the mitochondrial-targeted ROS scavenger MitoTEMPO, suggesting a mitochondrial source. However, ROS increase was not observed under combined treatment, and antioxidants (MitoTEMPO and NAC) did not prevent mitochondrial fragmentation (Figure S2), suggesting that ROS induced by VX-809 alone is not the main driver of the morphological changes due to combined treatment (VX-770 + VX-809 for 24 and 48 h).

Given the observed fragmentation, mitochondrial biogenesis may also be involved. Although markers of biogenesis such as PGC-1α/NRF1/2/TFAM were not assessed here, the increased mitochondrial fission observed under VX-809/VX-770 raises the possibility that impaired biogenesis contributes to mitochondrial dysfunction in CF. In addition, mitophagy and its efficiency can be influenced by the status of the autophagy machinery [31,97,98]. In various CF models, decreased autophagic activity has been reported, which could indirectly impair mitophagy and reduce the clearance of dysfunctional mitochondria [99,100]. This impairment has been linked to increased mTOR signaling, and the inhibition of the PI3K/AKT/mTOR pathway has been shown to enhance CFTR expression and stability. Future work evaluating these pathways under CFTR modulation will help to clarify its role in mitochondrial quality control.

Here, we demonstrate that combined treatment with VX-809 and VX-770 for 48 h resulted in a significant reduction (~50%) in mCx-I-III activity in the heterozygous CF IB3-1 cells. Despite this impairment, intracellular ATP levels or the ΔΨm remained unchanged, suggesting preserved bioenergetic homeostasis. This finding may reflect a compensatory adaptation, possibly involving enhanced reliance on alternative respiratory complexes, glycolysis [101,102], increased lactate dehydrogenase (LDH) activity [103], or enhanced TCA cycle input independent of mCx-I [104]. These adaptive responses may explain the preserved cell viability observed in the XTT assay, although this method also reflects cytosolic dehydrogenase activity [62]. Thus, despite impaired NADH–cytochrome c reductase (complex I–III) function, ATP production appeared to be maintained through compensatory mechanisms [105]. Future studies measuring oxygen consumption will help to define how, under CFTR modulator treatments, mitochondrial function adapts to reduced complex I-III enzymatic activity.

To analyze whether canonical mitochondrial fission and fusion pathways contributed to VX-809- and VX-770-induced fragmentation, we measured the protein expression of MFN1 and DRP1 by WBs (Figure 8). Morphological changes observed in IB3-1, S9 and C38 cells were not consistently associated with these proteins. In IB3-1 cells, DRP1 levels transiently increased (non-significantly) before detectable fragmentation, then returned to baseline, suggesting an early but reversible response not observed in S9 or C38 cells. Conversely, S9 cells showed an unexpected increase in MFN-1 expression despite fragmented mitochondria. These findings suggest that alternative, non-canonical mechanisms may be involved [106], such as mitophagy [107] or endoplasmic reticulum (ER)-dependent fission [108].

To further analyze whether chloride acted as a signaling molecule in VX-809/VX-770-induced mitochondrial changes [68,109,110], we measured the relative intracellular chloride concentration ([Cl^−^]_i_). Since no significant differences were detected in any cell lines, CFTR activity was further evaluated using an iodide (I^-^) efflux assay. Data suggest that in IB3-1 cells (carrying the heterozygous ΔF508/W1282X), treatment with VX-809/VX-770 for 48 h did not enhance I^-^ efflux, whereas S9 cells expressing functional CFTR showed higher baseline activity. The lack of correction likely reflects low ΔF508-CFTR expression, consistent with several reports suggesting that VX-809/VX-770 is ineffective in heterozygous CF [111]. Treatment with VX-809 (5 μM) for 48 h, in the absence of VX-770, in IB3-1 cells has been reported to restore CFTR activity [93], suggesting that the inclusion of VX-770 for 48 h could affect CFTR stimulation. Currently, in vitro research with CFTR modulators involves previous treatment with CFTR correctors (24–48 h) and a short incubation with the potentiator to stimulate CFTR [112,113], probably explaining our results.

Although lumacaftor/ivacaftor results in benefits in ΔF508-homozygous patients [56,114,115], its efficacy in heterozygous genotypes remains limited [49,116,117]. The therapeutic response to lumacaftor and ivacaftor has been widely reported to depend on the specific CFTR mutation variants [116,118,119,120,121,122]. The lack of functional rescue in IB3-1 cells agrees with previous studies showing that heterozygous mutant genotypes often have poor responsiveness to first-generation modulators such as VX-809, either alone or in combination with VX-770 [111]. These findings underscore the relevance of evaluating CFTR-independent effects of modulators, particularly in models with partial or absent pharmacological responses. Future experimental approaches using human primary cultures or cell lines carrying the homozygous ΔF508/ΔF508 genotype should be of value to evaluate mitochondrial morphology in the context of effective CFTR pharmacological correction.

Further studies should incorporate mitochondrial respirometry and structural analyses using the next-generation CFTR modulators such as the triple combination elexacaftor/tezacaftor/ivacaftor (ETI). Evaluating ETI in human primary bronchial epithelial cells from both homozygous and heterozygous ΔF508 models, where it has shown clinical efficacy [123], will provide a more clinically relevant context to investigate CFTR-dependent regulation of mitochondrial dynamics, the potential role of mitophagy, and the translational applicability of these findings.

## 5. Conclusions

In conclusion, our results suggest that combined treatment with CFTR modulators VX-809 and VX-770 induces mitochondrial fragmentation and reduces mitochondrial Cx-I-III activity without significantly affecting ΔΨm or ATP levels. Thus, mitochondrial morphology and ETC are modulated by CFTR modulator therapy. Although the clinical implications of these changes remain unclear, they may reflect either compensatory adaptations or unintended off-target effects of the compounds. The reduction in mCxI-III activity and increased mitochondrial fragmentation could arise from shared upstream mechanisms, such as altered ROS signaling, changes in membrane lipid composition by VX-770, or disruptions in Ca^2+^ homeostasis. Alternatively, these effects may represent independent consequences of CFTR modulation. This raises concerns about potential long-term effects of such therapeutic strategies on mitochondrial health and overall cellular function. In summary, these findings underscore the importance of incorporating mitochondrial function analyses into the preclinical evaluation of CFTR modulators. Such assessments could provide critical insights into their therapeutic efficacy and safety profiles, guiding the development of optimized CF therapies.

## Data Availability

The original contributions presented in this study are included in the article/Supplementary Material. Further inquiries can be directed to the corresponding author.

## Supplementary Materials

The following supporting information can be downloaded at: https://www.mdpi.com/article/10.3390/cells14201601/s1, **Figure S1.** Individual treatments with VX-809 or VX-770 do not alter the mitochondrial morphology of the heterozygous CF IB3-1 cells. (A) (a–c) Representative confocal microscopy images of IB3-1 cells individually treated with VX-809 (10 µM), VX-770 (0.1 µM) or vehicle (DMSO) and labelled with the fluorescent probe MitoTracker Orange. Mitochondrial morphology was analyzed using the MiNA and Micro-P tools. Color code for Micro-P mitochondria classification: small globular mitochondria (blue), simple tube (green, unbranched mitochondria), and branching tubes (purple, high connectivity). (B) (a–c) Bars and dots (replicates) showing the quantification of individual mitochondrial structures, mitochondrial network length, and the number of mitochondrial networks analyzed by MiNA. (d–f) Bars and dots (replicates) showing the quantification of small globes, simple tubes, and branching tubes analyzed by Micro-P. Data were analyzed by ANOVA one-way and Tukey post hoc test; **Figure S2.** Antioxidant treatments with MitoTEMPO and NAC failed to prevent mitochondrial fragmentation induced by combining VX-809 and VX-770 treatment in the heterozygous CF IB3-1 cells. (A) (a–c) Representative images observed through confocal microscopy of IB3-1 cells labelled with the fluorescent probe MitoTracker Orange and treated with VX-809 (10 µM) combined with VX-770 (0.1 µM) in presence or absence of the antioxidants MitoTEMPO (10 µM) and NAC (5 mM). Mitochondrial morphology was analyzed using the MiNA and Micro-P tools. Color code for Micro-P mitochondria classification: small globular mitochondria (blue), simple tube (green, unbranched mitochondria), and branching tubes (purple, high connectivity). (B) (a–c) Bars and dots (20–40 replicates) show the quantification of individual mitochondrial structures, mitochondrial network length, and the number of mitochondrial networks analyzed by MiNA. (d–f) Bars and dots (20–40 replicates) show the quantification of small globes, simple tubes, and branching tubes analyzed by micro-P. Data were analyzed by ANOVA one-way and Tukey post hoc test. ** *p* < 0.01, *** *p* < 0.001, **** *p* < 0.0001 indicate significant differences compared to the control group (DMSO).

## Abbreviations

The following abbreviations are used in this manuscript:

ATP	Adenosine triphosphate
BCA	Bicinchoninic acid
CF	Cystic fibrosis
CFTR	Cystic fibrosis transmembrane conductance regulator
CISD1	CISDGH iron sulfur domain-1
DCFH-DA	2′,7′-Dichlorodihydrofluorescein diacetate
DMSO	Dimethyl sulfoxide
DRP1	Dynamin-related protein 1
ETC	Electron transport chain
FCCP	Carbonyl cyanide-p-trifluoromethoxyphenylhydrazone
FDA	The United States Food and Drug Administration
FIS1	Mitochondrial fission 1 protein
HRP	Horseradish peroxidase
MAVS	Mitochondrial antiviral-signaling protein
mCx-I	Mitochondrial complex I
MFN1	Mitofusin 1
MFN2	Mitofusin 2
MiNA	Mitochondrial Network Analysis
OPA1	Optic atrophy 1
ROS	Reactive oxygen species
TMRE	Tetramethylrhodamine ethyl ester
ΔΨm	Mitochondrial membrane potential
