# Supplementary material for: Mitochondrial Fragmentation Induced by the CFTR Modulators Lumacaftor and Ivacaftor in Immortalized Cystic Fibrosis Cell Lines
Source: Cells. 2025 Oct 15;14(20):1601. doi: 10.3390/cells14201601 (PMC12564899; doi:10.3390/cells14201601)
Supplement: Supplementary file 1 [file cells-14-01601-s001.zip › cells-3723009-supplementary.pdf]

Mitochondrial Fragmentation induced by the CFTR Modulators Lumacaftor and Ivacaftor in Immortalized Cystic Fibrosis Cell Lines

Supplementary data

Camila Dib[1], Pablo A. Iglesias González[1], María de los Ángeles Aguilar[1], Tatiana Limpias del Valle[1], Nadia Nuñez Arno[1], Guillermo L. Taminelli[2], Analía G. Karadayian[1], Tomás A. Santa-Coloma[1] and Ángel G. Valdivieso[1,2,*]

[1] Laboratory of Cellular and Molecular Biology, Institute for Biomedical Research (BIOMED), School of Medical Sciences, Pontifical Catholic University of Argentina (UCA), and the National Scientific and Technical Research Council of Argentina (CONICET), Alicia Moreau de Justo 1600, Buenos Aires 1107, Argentina

[2] School of Engineering and Agrarian Sciences (FICA), Pontifical Catholic University of Argentina (UCA), Buenos Aires, Argentina

*Correspondence: angel_valdivieso@uca.edu.ar

Figure S1

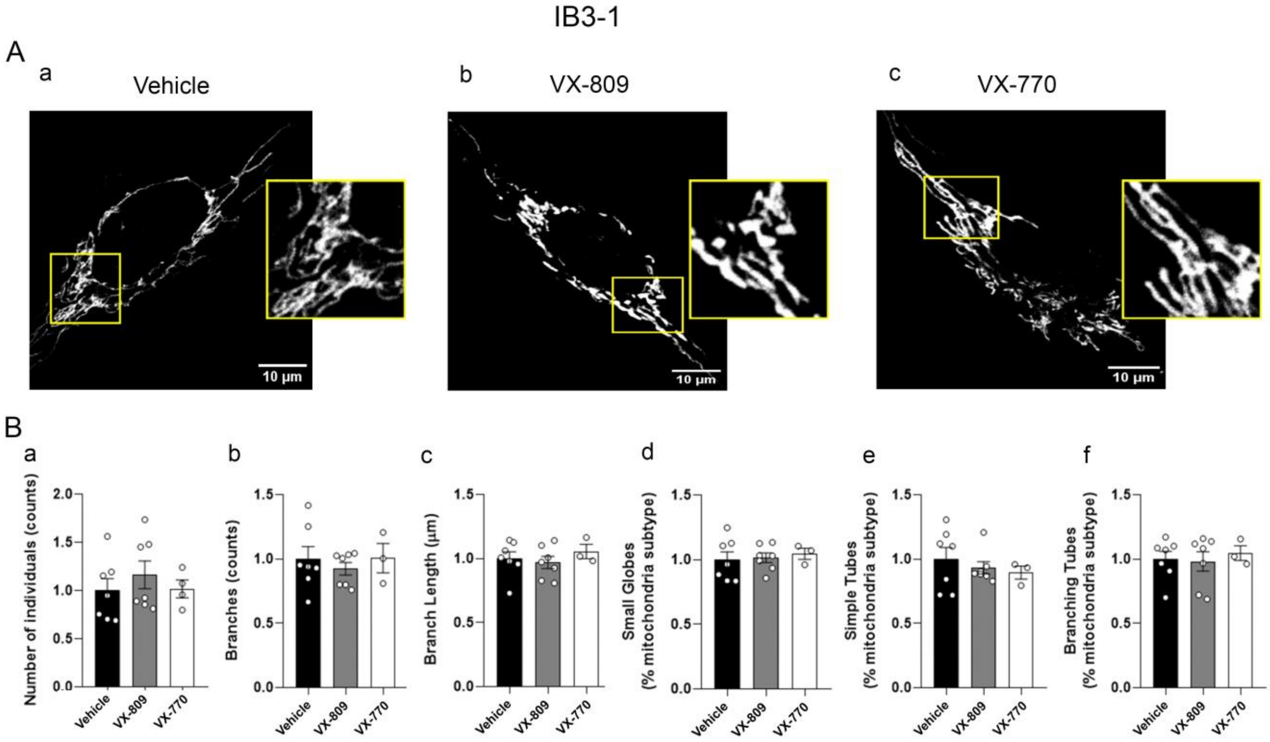

Figure S1. Individual treatments with VX-809 or VX-770 do not alter the mitochondrial morphology of the heterozygous CF IB3-1 cells. (A) (a-c) Representative confocal microscopy images of IB3-1 cells individually treated with VX-809 (10 µM), VX-770 (0.1 µM) or vehicle (DMSO) and labelled with the fluorescent probe MitoTracker Orange. Mitochondrial morphology was analyzed using the MiNA and Micro-P tools. Color code for Micro-P mitochondria classification: *small globular mitochondria* (blue), *simple tube* (green, unbranched mitochondria), and *branching tubes* (purple, high connectivity). (B) (a-c) Bars and dots (replicates) showing the quantification of individual mitochondrial structures, mitochondrial network length, and the number of mitochondrial networks analyzed by MiNA. (d-f) Bars and dots (replicates) showing the quantification of small globes, simple tubes, and branching tubes analyzed by Micro-P. Data were analyzed by ANOVA one-way and Tukey post hoc test.

Figure S2

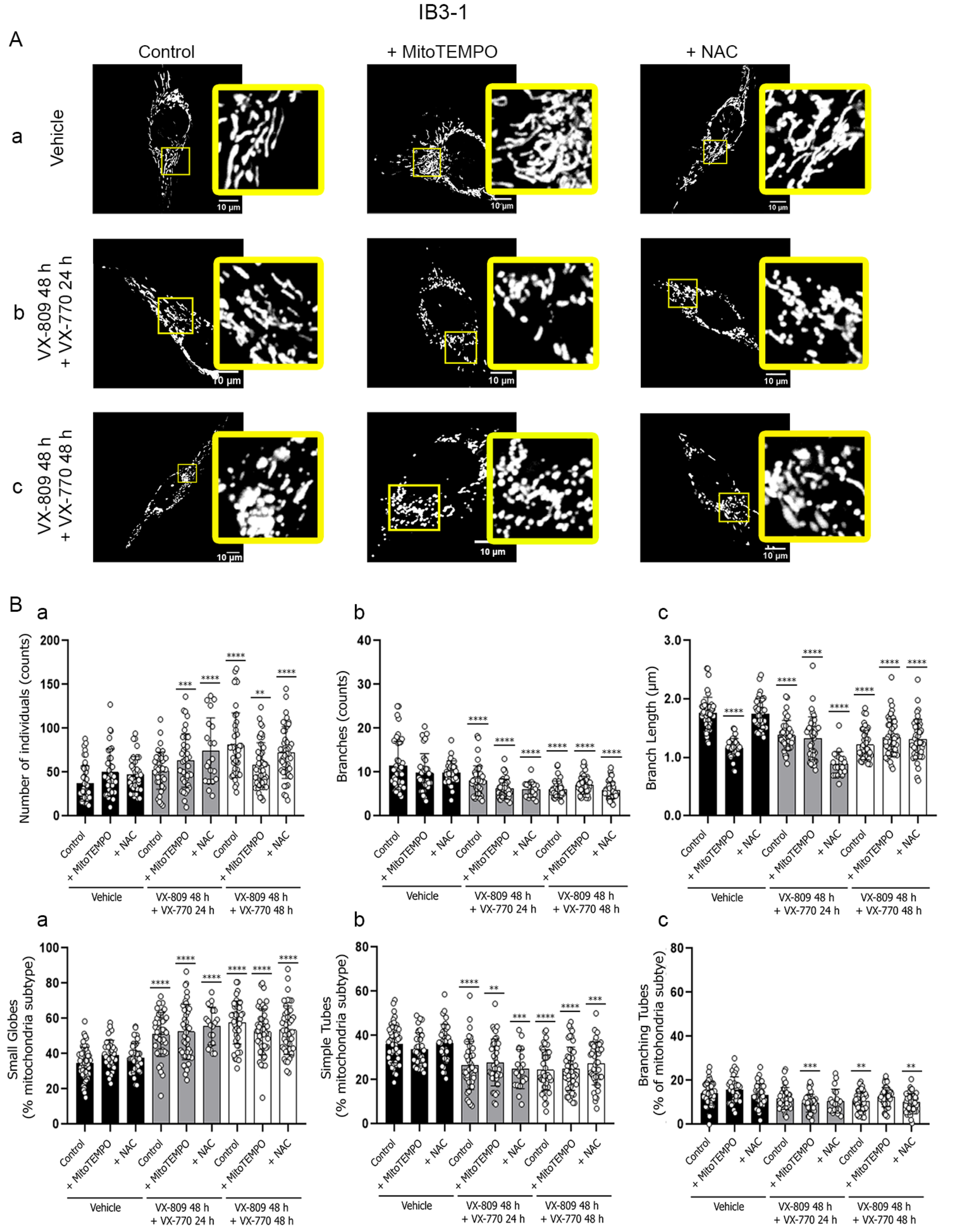

Figure S2. Antioxidant treatments with MitoTEMPO and NAC failed to prevent mitochondrial fragmentation induced by combining VX-809 and VX-770 treatment in the heterozygous CF IB3-1 cells. (A) (a-c) Representative images observed through confocal microscopy of IB3-1 cells labelled with the fluorescent probe MitoTracker Orange and treated with VX-809 (10 µM) combined with VX-770 (0.1 µM) in presence or absence of the antioxidants MitoTEMPO (10 µM) and NAC (5 mM). Mitochondrial

morphology was analyzed using the MiNA and Micro-P tools. Color code for Micro-P mitochondria classification: *small globular mitochondria* (blue), *simple tube* (green, unbranched mitochondria), and *branching tubes* (purple, high connectivity). (B) (a-c) Bars and dots (20-40 replicates) show the quantification of individual mitochondrial structures, mitochondrial network length, and the number of mitochondrial networks analyzed by MiNA. (d-f) Bars and dots (20-40 replicates) show the quantification of small globes, simple tubes, and branching tubes analyzed by micro-P. Data were analyzed by ANOVA one-way and Tukey post hoc test. * $p < 0.05$, ** $p < 0.01$, *** $p < 0.001$, **** $p < 0.0001$ indicate significant differences compared to the control group (DMSO).
